# Weight-bearing computed tomography in knee pathologies: current evidence and future perspectives

**DOI:** 10.3389/fsurg.2026.1769099

**Published:** 2026-02-19

**Authors:** Andre Giardino Moreira da Silva, Renata Vidal Leão, Carlos Felipe Teixeira Lobo, Enzo Tunala Mendonça, Camilo Partezani Helito

**Affiliations:** 1 Instituto de Ortopedia e Traumatologia, Hospital das Clínicas HCFMUSP, Faculdade de Medicina, Universidade de São Paulo, São Paulo, Brazil; 2 University of Iowa Hospitals and Clinics, Iowa City, IA, United States; 3 Faculdade de Medicina, Universidade de São Paulo, São Paulo, Brazil; 4 Hospital Sírio Libanês, São Paulo, Brazil

**Keywords:** anterior cruciate ligament, knee alignment, knee osteoarthritis, knee pathology, patellofemoral, total knee arthroplasty, weight-bearing CT

## Abstract

Weight-bearing computed tomography (WBCT) provides three-dimensional (3D), high-resolution imaging with patients in either a unipodal or bipodal stance, allowing visualization of dynamic joint alterations that might be missed in conventional radiographs or in non–weight-bearing exams, such as conventional computed tomography (CT) or magnetic resonance imaging (MRI) scans. Multiple lines of research are exploring its application for the evaluation of knee osteoarthritis, knee ligamentous instability, malalignment syndromes, patellofemoral disorders, and postoperative assessment following total knee arthroplasty. Despite its growing clinical utility, the development of standardized imaging protocols, broader accessibility, and integration with advanced image-analysis tools remain important areas for further progress. This review summarizes the current evidence supporting the clinical applications of WBCT in knee assessment and discusses future directions aimed at optimizing its role in personalized musculoskeletal care.

## Introduction

The assessment of knee pathologies has long relied on conventional imaging modalities such as plain radiography, magnetic resonance imaging (MRI), and standard computed tomography (CT). While each of these techniques provides valuable diagnostic information, they also have inherent limitations—particularly in accurately assessing joint alignment under physiological loading conditions, as they fail to replicate the dynamic stresses experienced by the knee during daily activities. Plain radiographs, although performed under weight-bearing conditions and widely used in clinical practice to evaluate joint space narrowing and overall knee alignment, provide only a two-dimensional representation of the knee's complex three-dimensional structure. As a result, they are susceptible to positioning errors and image superimposition, and fail to adequately assess rotational abnormalities or subtle multiplanar deformities (1).

In this context, weight-bearing computed tomography (WBCT), which has been increasingly employed for the evaluation of foot and ankle pathologies, has seen a growing body of literature exploring its application in knee pathologies in recent years, as advances in technology have enabled the scanning of the knees and hips (2). Current lines of research are investigating its potential roles in evaluating knee osteoarthritis, ligamentous instability, malalignment syndromes, patellofemoral disorders, and in postoperative assessment following procedures such as high tibial osteotomy or total knee arthroplasty. As orthopedic practice increasingly shifts toward personalized and biomechanics-based approaches, WBCT represents a significant step forward in imaging assessment. By bridging the gap between static imaging and functional evaluation, it enhances orthopedic surgeons’ ability to characterize biomechanical dysfunctions and to optimize both surgical planning and overall rehabilitation strategies. This article aims to present a narrative review of the current applications and emerging evidence regarding the use of WBCT in the evaluation of knee pathologies, with particular emphasis on its potential to enhance diagnostic accuracy and guide therapeutic decision-making.

## Technical aspects and scanning protocols

Most contemporary WBCT systems used for the knee joint are based on cone-beam computed tomography (CBCT) technology, which employs a flat-panel detector and a low-power x-ray source rotating around the patient in an upright position while the joint of interest is imaged under weight-bearing conditions (3). The systems most commonly used in musculoskeletal research include the Planmed Verity scanner (Planmed, Helsinki, Finland), the Carestream OnSight 3D Extremity System (Carestream Health, Rochester, NY, USA), and CurveBeam scanners (CurveBeam, Warrington, PA, USA), such as PedCAT, PedCAT Premium, HiRise, and LineUp (2, 4, 5). Additionally, the prototype TSX-401R (Canon Medical Systems, Otawara, Japan), a multidetector CT system rather than a cone-beam CT platform (6), is capable of imaging patients in a standing position and has also been utilized in extremity imaging studies (7). These systems differ substantially in their operational capabilities and limitations, including variations in field of view (FOV), differences in patient positioning possibilities within the scanner, and the ability to perform unilateral or bilateral acquisitions, as well as whether imaging is limited to the foot and ankle or extends to the knee and/or hip. Some scanners are restricted to unilateral acquisitions (Planmed), whereas others are capable of bilateral imaging but remain limited to distal joints such as the ankles and feet (PedCAT, PedCAT Premium). Other systems allow bilateral imaging of both the ankles and knees (OnSight, LineUp). Modern systems, such as the CurveBeam HiRise, enable weight-bearing image acquisition up to the hip and pelvis, allowing bilateral imaging of the hips, knees, and feet without patient repositioning.

With respect to acquisition protocols, published studies have explored a wide range of patient positions tailored to different pathological conditions, including protocols with varying knee flexion angles (ranging from 0°, 20°, 30°, 60°, to 120°), single-leg and double-leg weight-bearing, and neutral, internal, and external knee rotation. To date, no standardized, pathology-specific acquisition protocol has been defined in the literature. The specific characteristics of each published protocol in the context of different knee pathologies will be discussed further in this review.

Regarding radiation dose delivered during weight-bearing cone-beam CT (WBCT) scans of the knee, dosimetry studies have demonstrated lower radiation exposure compared with conventional multidetector CT (MDCT), while remaining higher than that of standard plain radiographs (8, 9). In a phantom dosimetry study using anthropomorphic knee models, the effective dose of an extremity CBCT scan was approximately 12.6 µSv, compared with 27–48 µSv for conventional multislice CT protocols, corresponding to a two- to four-fold dose reduction with cone-beam CT for knee imaging (8). Conventional radiography (anteroposterior and lateral projections), in contrast, produced effective doses of approximately 1.2–1.8 µSv, thereby representing the lowest radiation exposure (8). In a systematic review comparing the effective radiation dose of extremity cone-beam CT (CBCT) with other imaging modalities, Mason et al. (9) reported that most studies demonstrated that CBCT delivers lower radiation doses than multislice CT, with effective doses typically less than half those of multislice CT.

## Weight-bearing CT in the assessment of knee pathologies

### Patellofemoral pathologies

Patellofemoral disorders, including patellofemoral pain syndrome and patellar instability, are multifactorial conditions associated with mechanical overload, lower-limb malalignment, impaired neuromuscular control, and abnormal patellar tracking. In current clinical practice, parameters used to evaluate patellar maltracking are typically assessed with static, non–weight-bearing imaging modalities such as plain radiographs, conventional computed tomography, and magnetic resonance imaging. However, preoperative assessments performed without weight-bearing and without active muscle contraction may not accurately reproduce the physiological conditions and joint relationships in which these pathologies occur, affecting both patellofemoral and tibiofemoral alignment and potentially leading to less accurate quantification of the anatomical factors relevant to the pathology.

**Figure 1:**
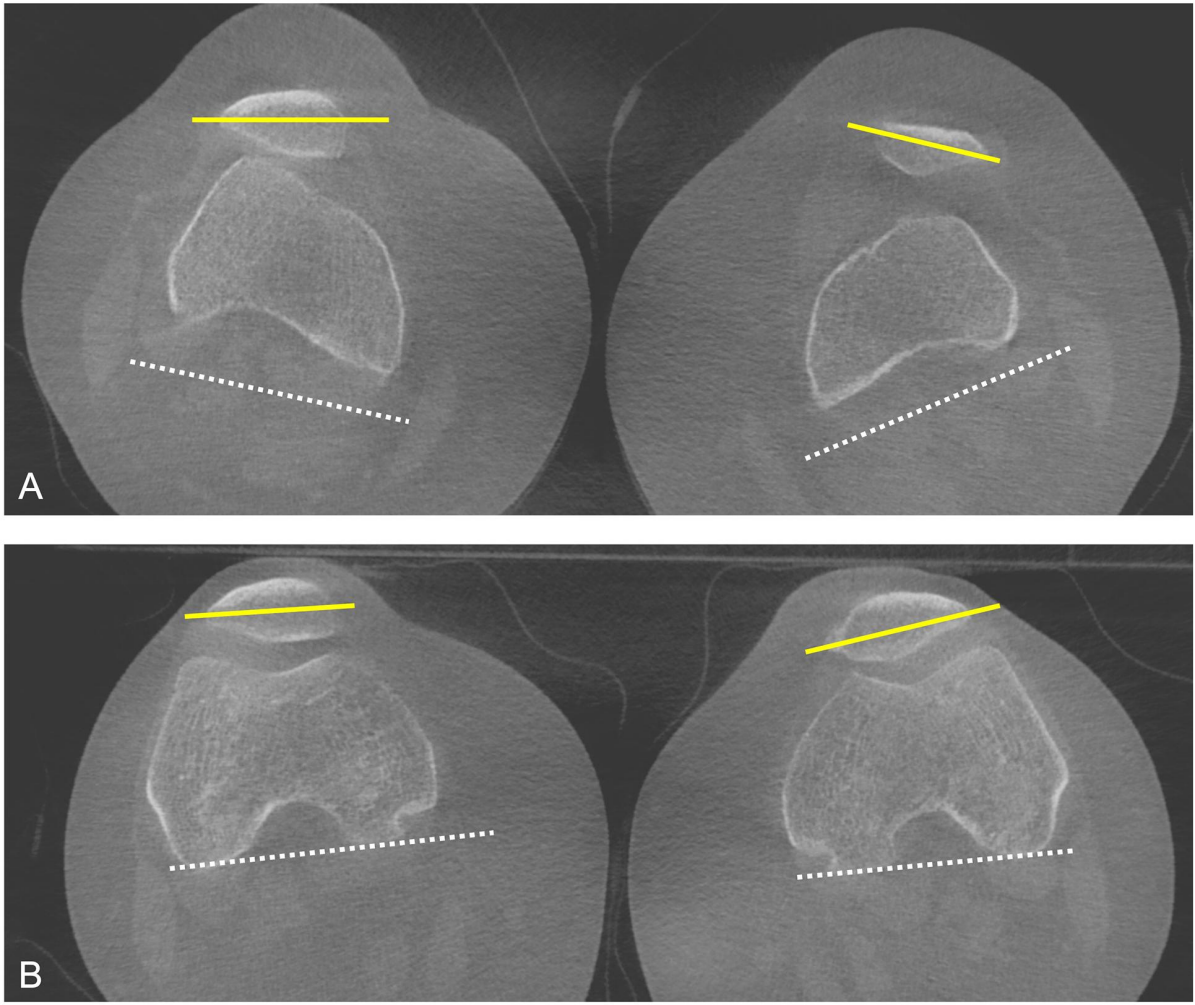
Patient with patellofemoral instability. **(A)** Weight-bearing CT in full extension demonstrates increased lateral patellar tilt and lateral subluxation. **(B)** At 30° of knee flexion, WBCT demonstrates patellar reduction with restoration of patellofemoral alignment, reflecting the engagement of the patella within the trochlear groove.

**Figure 2:**
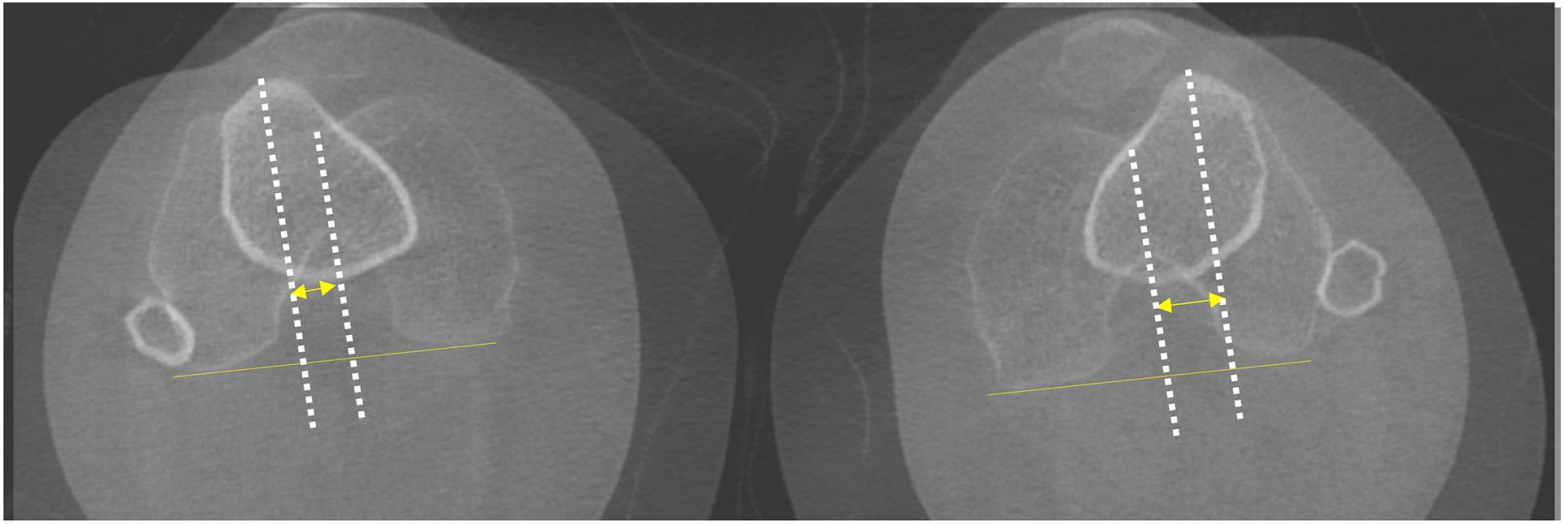
Patient with left knee patellofemoral instability. WBCT images acquired in extension allow bilateral assessment of the TT–TG distance, demonstrating an increased TT–TG value on the left side.

**Figure 3:**
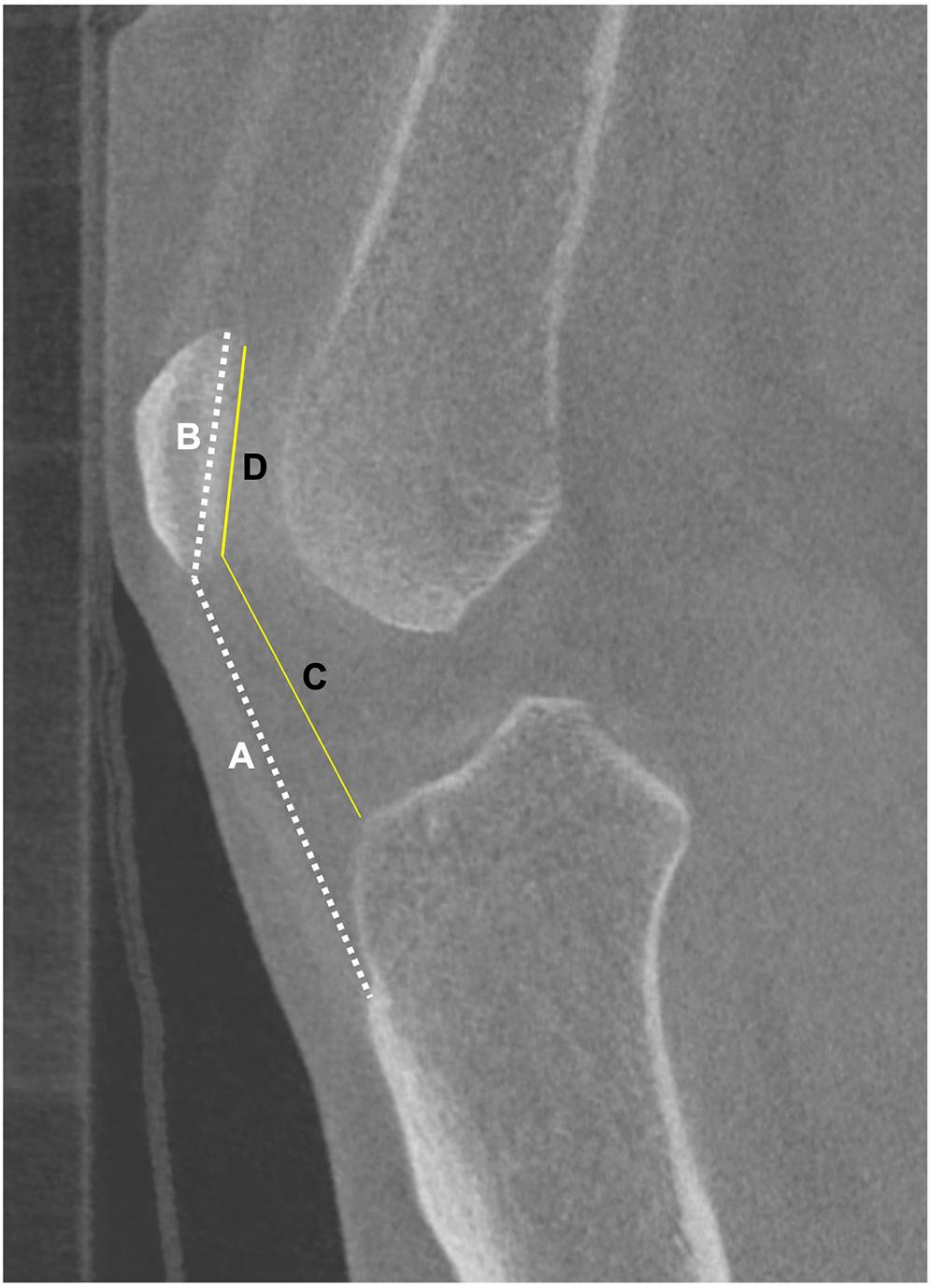
Patellar height assessment on WBCT at 30° of knee flexion. A/B: Insall–Salvati index. C/D: Caton–Deschamps index.

Common parameters for evaluating patellar tracking include the tibial tubercle–trochlear groove (TT–TG) distance (10), patellar tilt angle (10), and patellar height indices such as the Insall–Salvati (11) and Caton–Deschamps (12) ratios, and the behavior of these parameters under weight-bearing and non–weight-bearing conditions has been studied by several authors (Figures 1–3). Hirschmann et al. (13) compared conventional supine CT with upright weight-bearing CT (WBCT), both in full extension, and observed load-dependent differences, including a shift from external to internal femorotibial rotation (2.7° ± 5.1 to −0.4° ± 7.7; *p* = 0.009), a reduction in TT–TG distance (13.8 ± 5.1 mm to 10.5 ± 5.0 mm; *p* = 0.008), and decreased lateral patellar tilt (15.6° ± 6.7 to 12.5° ± 7.7; *p* = 0.011), emphasizing that supine CT may not accurately represent physiological weight-bearing knee alignment. Marzo et al. (14), in turn, evaluated patellofemoral alignment in 20 patients with lateral patellar instability using both a 30° knee-flexion, upright WBCT scan and a conventional supine CT scan. On the 30° flexion WBCT scan, the mean tilt angle was 18.2° ± 11.6° compared with 28.1° ± 7.1° on the conventional CT (*P* < .0001), the mean congruence angle was 3.0° ± 30.1° compared with 26.7° ± 18.1° on the conventional CT (*P* = .0002), and the TT-TG offset distance averaged 12.3 ± 6.3 mm compared with 20.1 ± 4.2 mm on the conventional CT (*P* < .0001). Good interrater reliability was found for tilt angle, congruence angle, and TT-TG offset on both conventional and CBCT scans (ICC range, 0.79–0.96). In a subsequent study, Marzo et al. (15) evaluated axial alignment using WBCT with the knee at 30° of flexion in 20 healthy volunteers with no history of knee complaints and found a mean TT-TG offset of 2.7 mm, which is lower than the historically reported normal range, and demonstrated good to moderate interrater reliability and good interrater reliability.

Regarding the influence of flexion angle on knee alignment, Hirschmann et al. (16) evaluated CT scans of the knee at 0°, 30°, and 60° of flexion in the upright weight-bearing position and found that, with increasing flexion, the TT–TG distance decreased (0°/30°/60° flexion: mean, 11.1 mm ± 3.7/3.6 mm ± 3.2/2.6 mm ± 5.5), whereas internal tibiofemoral rotation increased (0°/30°/60° flexion: mean, 1.2° ± 5.1/10.7° ± 4.2/13.2° ± 5.2). These differences were statistically significant between 0° and 30° and between 0° and 60°, but not between 30° and 60°. This is likely explained by the screw-home mechanism, which consists of approximately 15° of external rotation of the tibia relative to the femur during the final 20° of extension in normal knee kinematics (17), resulting in a substantial change in femur–tibia positioning between 0° and 30° of flexion. Buzzatti et al. (18), in turn, investigated patellar kinematics in 21 healthy adults (42 knees) using a horizontal dynamic CT system that simulated weight-bearing via a sliding bed attached to a counter-weight and pulley system calibrated to 42% of each subject's body weight. During a squat-like flexion–extension motion (0°–30° of knee flexion), the tibial-tuberosity–trochlear-groove (TTTG) distance, bisect offset (BO), and lateral patellar tilt (LPT) all decreased progressively in the eccentric (flexion) phase (TTTG by 6.9 mm, BO by 12.6%, LPT by 4.3°) and then increased again in the concentric (extension) phase (TTTG +6.8 mm, BO +14.1%, LPT +4.3°).

Regarding postoperative evaluation of patellar instability, Lullini et al. (19) assessed patellofemoral alignment in 17 patients at 60-month follow-up after surgical treatment for recurrent patellar dislocation—including isolated MPFL reconstruction and associated with tibial tubercle osteotomy, with or without trochleoplasty—using both WBCT and conventional CT. They found that the TT–TG offset was significantly smaller on WBCT (9.9 ± 5.3 mm) compared with conventional CT (15.9 ± 4.9 mm; *p* < 0.001). WBCT also demonstrated superior measurement consistency (ICC 0.80–0.94 vs. 0.52–0.78), suggesting that conventional CT may overestimate TT–TG and that WBCT provides a more reliable pre and postoperative assessment of patellofemoral instability.

On the other hand, Sasaki et al. (20) evaluated the TT–TG distance and the Insall–Salvati ratio using full-extension upright CT in 26 healthy volunteers, monitoring foot-pressure distribution with a pressure-mat system: one limb bore full weight, while the contralateral limb provided only minimal support (∼2 kg, defined as non–weight-bearing). The Insall–Salvati ratio remained similar between conditions (0.98 ± 0.15 vs. 1.00 ± 0.12; *p* = 0.29), whereas the TT–TG distance was significantly greater under weight-bearing compared with the upright non–weight-bearing side (20.3 ± 3.9 mm vs. 12.3 ± 4.7 mm; *p* < 0.001). Although Sasaki et al.'s study (20) was limited by comparing contralateral limbs, their finding that the TT–TG distance decreases under upright non–weight-bearing conditions contrasts with the results of other studies (13, 14, 19, 21), in which TT–TG distance was greater on supine non–weight-bearing CT. These discrepancies suggest that knee alignment is influenced not only by axial load, but also by body posture (upright vs. supine) and by the distribution of load between the lower limbs, both of which may substantially affect TT–TG measurements. The increased load during single-leg stance in patients with weakness of the hip abductors and external rotators - a condition that is common even in asymptomatic individuals - may cause the femur to drift into internal rotation during single-limb support (22). This relative internal rotation of the femur with respect to the tibia may, in turn, lead to an increase in the TT–TG distance. This apparently contradictory finding underscores the need for standardized acquisition protocols and measurement techniques in studies involving WBCT.

Chen et al. (21) conducted a prospective study comparing patients with recurrent patellar dislocation and control subjects using both WBCT and conventional non weightbearing CT scans to assess patellofemoral alignment and its relationship with anatomic factors. In both groups, the Insall-Salvati ratio and the TT-TG offset were lower in WBCT compared with conventional non-weight-bearing CT (NWBCT) (recurrent patellar dislocation/control: *p* = 0.001/*p* < 0.001 and *p* = 0.006/*p* < 0.001, respectively), while the bisect offset index was higher in the WBCT (recurrent patellar dislocation/control: *p* < 0.001/*p* < 0.001). In the control group, the Blackburne-Peel ratio and the Caton-Deschamps ratio were lower with weightbearing compared with nonweightbearing (*p* = 0.01 and *p* = 0.007, respectively). The anatomic factor most strongly correlated with recurrent patellar dislocation during weightbearing was the bisect offset index [r = 0.73 (95% CI 0.65–0.79); *p* < 0.001], whereas during nonweightbearing, sulcus depth was the strongest predictor [r = −0.70 (95% CI-0.78 to −0.59); *p* < 0.001]. Receiver Operating Characteristic (ROC) analysis showed that during weightbearing, the bisect offset index had the best diagnostic ability for recurrent patellar dislocation [Area Under the Curve (AUC) 0.93 (95% CI 0.89–0.97)], while sulcus depth was the most predictive factor in the non-weight-bearing state [AUC 0.91 (95% CI 0.85–0.96)].

These findings suggest that weight-bearing imaging may more accurately identify patients who could benefit from tibial tuberosity medialization and/or distalization, indicating that new weight-bearing–specific cutoff values should be established and validated for WBCT exams.

### Knee osteoarthritis

Weight-bearing CT has become a valuable tool in the assessment of knee osteoarthritis (OA) because it provides three-dimensional imaging under physiological load, revealing structural changes such as joint space width (JSW), meniscal extrusion, subchondral bone morphology, cartilage loss, and malalignment that are often underestimated on standing radiographs or non-weight-bearing CT or MRI.

**Figure 4:**
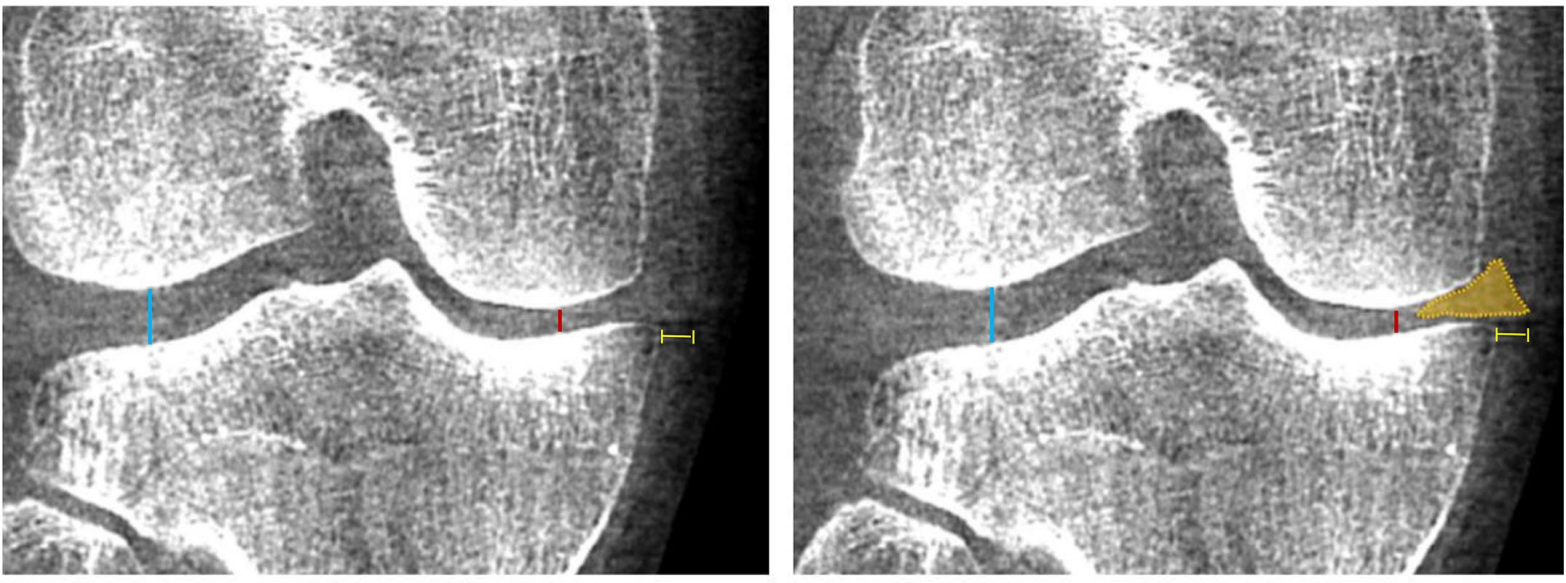
WBCT of a patient with medial compartment osteoarthritis of the right knee, demonstrating the joint space width of the medial compartment (red line) and lateral compartment (blue line), as well as extrusion of the medial meniscus (yellow line).

**Figure 5:**
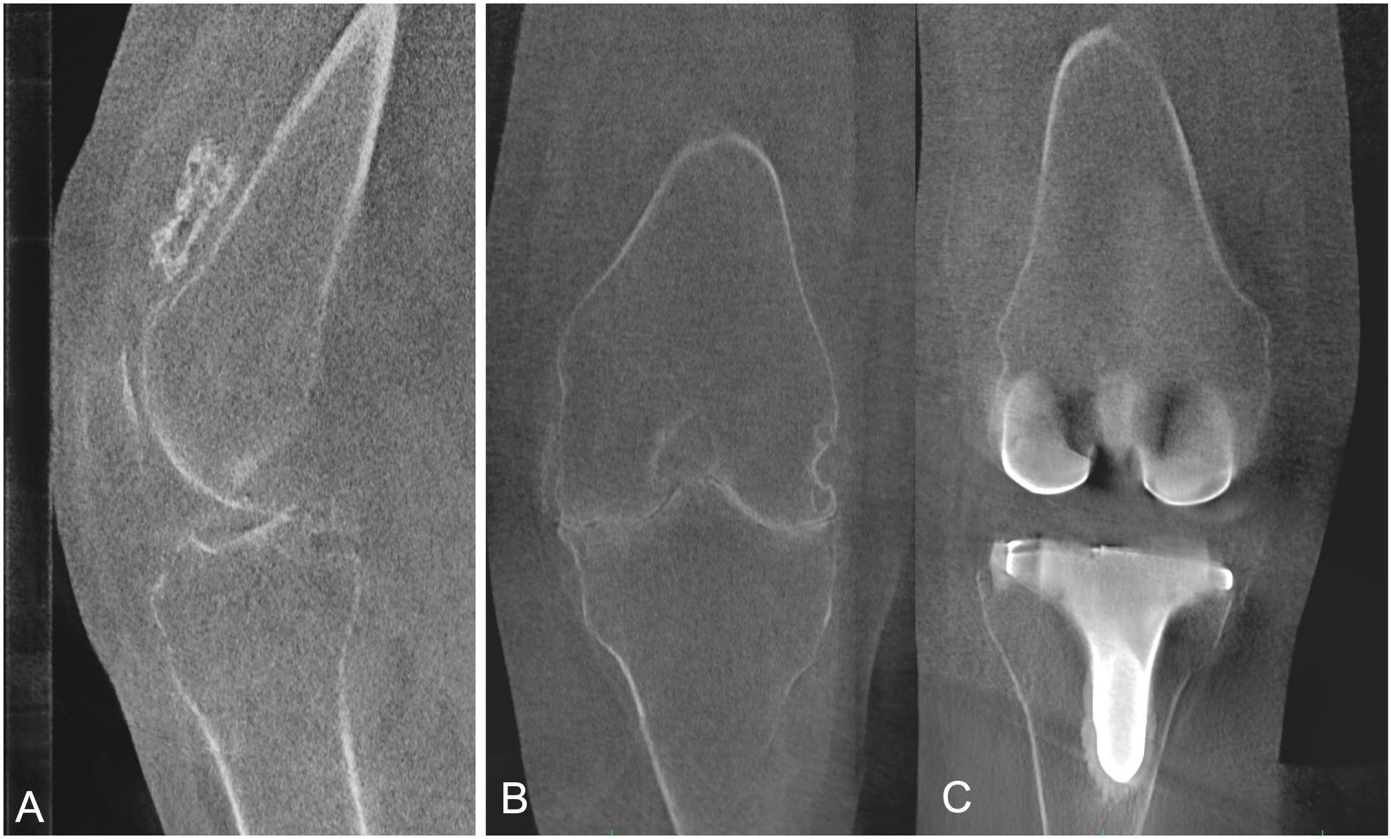
Weight-bearing CT in a patient with severe knee osteoarthritis. **(A)** Sagittal WBCT image shows joint bodies within the suprapatellar recess. **(B)** Coronal WBCT image demonstrates complete collapse of the medial joint space under load, consistent with advanced degenerative disease. **(C)** The contralateral knee arthroplasty is also visualized, without evidence of hardware complications.

Joint space width measurement (Figures 4, 5) has long been key parameter to monitoring OA progression, yet conventional radiographs are limited by projectional distortion and inconsistent patient positioning. In a comparative study of standing fixed-flexion posteroanterior radiographs and WBCT acquired in the identical position, Segal et al. (23) demonstrated that, despite a strong correlation between radiographic and WBCT medial JSW measurements, radiographs systematically overestimated the minimum JSW, likely due to overlapping anatomy that obscures the true joint margins. At the most medial tibiofemoral locations, the mean overestimation was approximately 2.0 mm, underscoring the superior accuracy of WBCT in capturing true load-bearing joint space narrowing. Fritz et al. (24) evaluated WBCT, NWBCT, and standing radiographs to quantify femorotibial joint space width and found that mean JSW was significantly narrower on WBCT than NWBCT in both the medial (4.7 vs. 5.1 mm, *p* = 0.028) and lateral compartments (6.3 vs. 6.8 mm, *p* = 0.008), and that minimal JSW on standing radiographs (medial: 3.1 mm; lateral: 5.8 mm) was significantly wider than on both CT modalities (1.8 mm and 2.9 mm, all *p* < 0.001). WBCT also detected more bone-on-bone apposition, an indicator of high grade or full thickness opposing cartilage loss, than NWBCT (25% vs. 10%, *p* = 0.008) and weight bearing radiographs (25% vs. 8%, *p* = 0.012) (24). Additionally, longitudinal data further demonstrated the superiority of WBCT over plain radiographs in detecting knee OA progression and joint space narrowing. Segal et al. (25) followed 265 patients for 24 months and found that WBCT was significantly more responsive for maximal JSW changes in the central medial and lateral femoral and tibial subregions (*p* < 0.001) and for mean JSW changes in the central medial femoral (*p* < 0.001) and tibial (*p* < 0.002) subregions, allowing earlier detection of structural deterioration.

Regarding clinical-radiological correlations, Kothari et al. (26) assessed the cross-sectional association between JSW measured by WBCT and self-reported pain and physical function using the Western Ontario and McMaster Universities Osteoarthritis Index (WOMAC) in 528 knees. They found that patients with a larger medial tibiofemoral area with JSW <2.0 mm experienced greater functional limitations (*p* = 0.02 for the highest vs. lowest tertile). In contrast, in a longitudinal study, Yoshikawa et al. (27) evaluated whether JSW measured on WBCT better predicted 24-month worsening of knee pain and function compared with JSW measured on standing fixed-flexion posteroanterior radiographs. Among 425 knees, WBCT did not significantly outperform radiography in predicting minimal clinically important worsening for WOMAC pain, WOMAC function, 20-meter walk, or sit-to-stand. Despite similar predictive performance between WBCT and plain radiographs, likely due to the weak correlation between clinical and radiographic worsening in knee osteoarthritis (28), the authors highlight that WBCT offers enhanced imaging capabilities that make it valuable for osteoarthritis assessment.

The ability to assess meniscal extrusion under physiological load represents another advantage of WBCT, enabling direct and reliable evaluation of a key marker of meniscal dysfunction with broad clinical relevance (Figure 4). Although it is also possible to perform this assessment using ultrasound, WBCT is not examiner-dependent and provides numerous other additional pieces of information. Thawait et al. (29) evaluated non–weight-bearing (NWB) and weight-bearing (WB) CT scans of patients with medial compartment OA and found a significant reduction in joint space width (2.1 mm vs. 1.5 mm, *p* = 0.016) and an increase in meniscal extrusion (6.9 mm vs. 8.2 mm, *p* = 0.018), whereas no significant differences were observed in these measures among non-OA patients. Segal et al. (30) evaluated the ability of WBCT vs. conventional MRI to detect meniscal extrusion in 56 participants from the Multicenter Osteoarthritis Study (MOST) and found that MRI missed 36% of medial meniscal extrusions detected by WBCT using a sharp kernel with 0.3 mm coronal slices, which showed higher extrusion scores than MRI in 66% of cases, while lateral extrusion was higher on WBCT in 10.7% of knees. Thus, WBCT also provides a more reliable evaluation of meniscal extrusion, which is often underestimated on supine MRI because the meniscus re-centers when unloaded. Under weightbearing, however, WBCT consistently shows greater extrusion, reflecting early meniscal insufficiency that contributes to cartilage overload and OA progression.

In addition to joint space narrowing and meniscal extrusion, osteophytes and subchondral cysts are important features of osteoarthritis that should also be routinely assessed. Segal et al. (31) evaluated the diagnostic performance of standing cone-beam CT of the knee for detecting osteophytes and subchondral cysts in 20 participants, using MRI as the reference standard. WBCT demonstrated significantly higher sensitivity and accuracy than fixed-flexion radiographs for osteophytes (sensitivity 93% vs. 60%, accuracy 95% vs. 79%; *p* < 0.004) and subchondral cysts (sensitivity 100% vs. 10%, accuracy 99% vs. 94%; *p* < 0.001), highlighting WBCT as a more reliable method than conventional radiography for detecting key structural features of knee osteoarthritis.

In the evaluation of the patellofemoral joint, Segal et al. (32) assessed the diagnostic performance of WBCT vs. lateral radiographs for detecting patellofemoral osteoarthritis features in 60 knees, using MRI cartilage damage as the reference standard. WBCT demonstrated markedly higher sensitivity (0.85–0.97 vs. 0.47–0.57) and accuracy (0.85–0.92 vs. 0.48–0.57) than radiographs for nearly all parameters (*p* < 0.001), along with moderate-to-strong agreement with MRI. By providing superior detection of patellofemoral OA at radiation levels comparable to radiographs, WBCT has the potential to substantially improve assessment of patellofemoral joint degeneration. Moreover, the role of WBCT arthrography is also being investigated and has shown greater precision in evaluating cartilage defects compared with MRI. When using WBCT arthrography as the reference standard, MRI sensitivity for detecting cartilage lesions ranged widely (0%–79%), and chondral lesions were not visualized or were underscored in 3.1–33.8% of cases depending on the compartment examined (33). Thus, while excellent for soft-tissue visualization, MRI may miss or undergrade chondral lesions relative to WBCT arthrography.

Recent research has identified increased internal tibial rotation under weightbearing as a potential early biomechanical marker of knee OA. Kaneda et al. (7) evaluated 45 osteoarthritic knees using supine CT and upright WBCT to quantify three-dimensional alignment changes across Kellgren–Lawrence (KL) grades and found significantly greater weight-bearing–induced deformities with increasing OA severity, including flexion changes (KL 1: 0.04 ± 1.91° vs. KL 4: 4.08 ± 4.36°, *p* = 0.020), adduction/varus changes (KL 1: 0.40 ± 0.62° vs. KL 4: 3.03 ± 2.16°, *p* < 0.001), and especially tibial internal rotation (KL 1: 0.34 ± 1.26° vs. KL 2: 2.95 ± 1.44°, KL 3: 3.60 ± 2.25°, KL 4: 3.88 ± 2.00°; *p* = 0.008 to <0.001). Notably, significant tibial internal rotation differences emerged earlier than changes in flexion or adduction, leading the authors to propose internal rotation as a potential early marker for OA detection.

Beyond degenerative imaging features such as JSW, meniscal extrusion, osteophytes, and subchondral cysts, a three-dimensional assessment of joint space provides insights into knee osteoarthritis that conventional imaging cannot capture. Turmezei et al. (34) demonstrated that WBCT-based joint space mapping (3D-JSM)—defined as the distance between femoral and tibial subchondral bone surfaces—reliably detects subtle JSW variations in load-bearing regions of the medial and lateral compartments. In a larger cohort, they further demonstrated that 3-D multiparametric analysis for joint space mapping and cortical bone mapping measures—including subchondral plate thickness, endocortical thickness, and trabecular attenuation— enhances the detection of structural changes (35). This analysis revealed that the central-to-posterior medial tibiofemoral joint space narrows by up to 0.5 mm and tibial trabecular attenuation increases by up to 50 units with each increment in Kellgren & Lawrence grade, with an even broader distribution of significant findings when parameters are combined (*p* < 0.05) (35). These studies validate the clinical utility of WBCT-derived 3D-JSM, demonstrating its correlation with OA severity and alignment changes and suggesting that it may enable more sensitive monitoring of osteoarthritis progression.

In summary, WBCT enables evaluation of joint space width, meniscal extrusion, cartilage-related changes, subchondral structures, and functional alignment, including early rotational abnormalities. While MRI remains the gold standard for detailed assessment of soft tissue injuries, such as meniscal tears, osteochondral lesions, ligament degeneration, and overload-related subchondral bone lesions, WBCT provides superior assessment of joint alignment and load-bearing–related conditions, including joint space width and meniscal extrusion. Rather than replacing MRI or plain radiographs, WBCT complements these imaging modalities by offering a three-dimensional evaluation of the knee under physiological loading conditions.

### ACL instability

**Figure 6:**
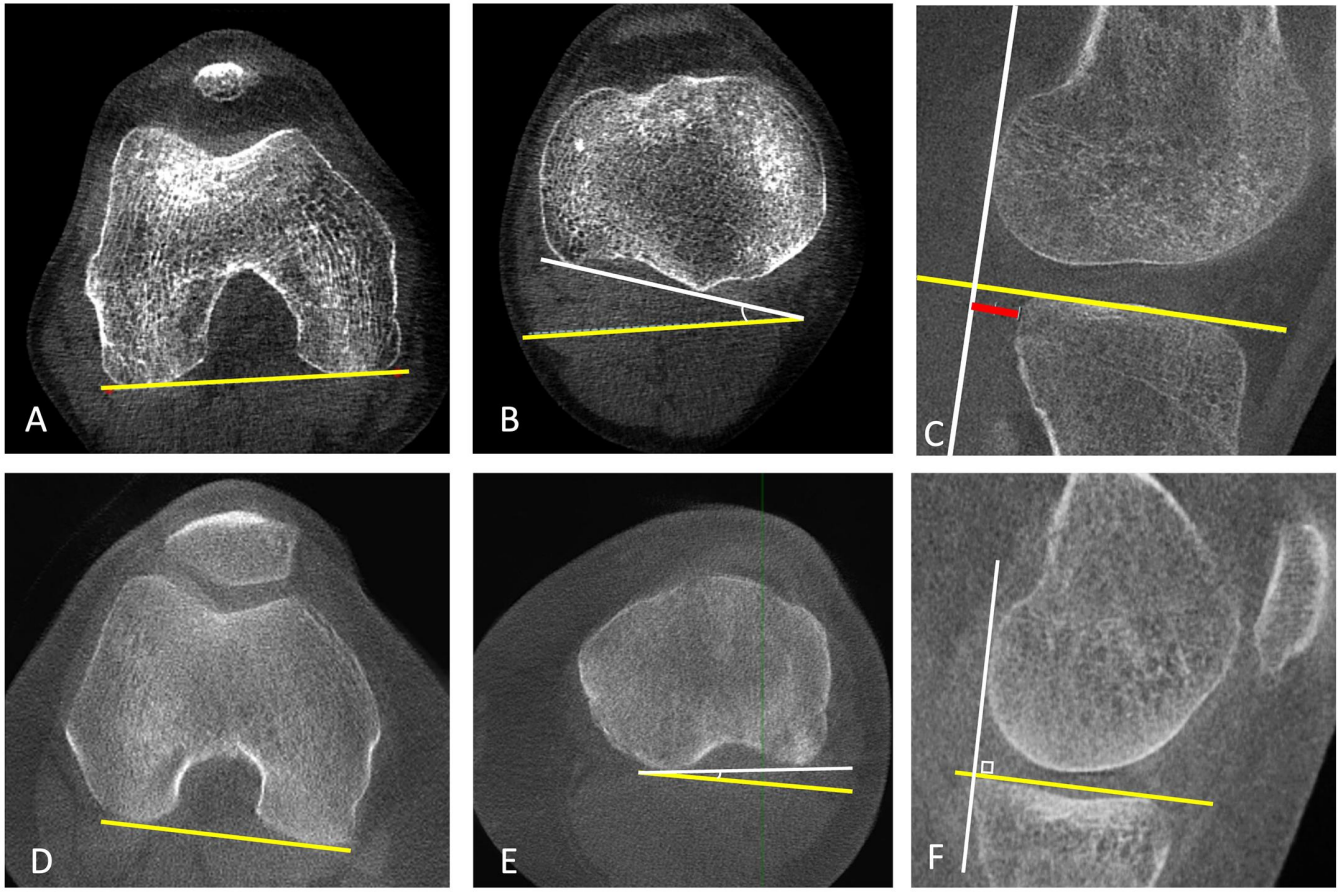
WBCT in a patient with a unilateral ACL tear of the right knee **(A–C)** and an intact ACL in the contralateral left knee **(D–F)**. Axial WBCT images demonstrate increased internal tibial rotation on the ACL-injured side **(A,B)** compared with the uninjured side **(D,E)**. Sagittal WBCT image of the injured knee **(C)** shows increased anterior tibial translation relative to the femur, whereas the uninjured side demonstrates absence of anterior tibial translation **(F)**.

In the assessment of ACL tears, conventional clinical tests such as the Lachman and pivot-shift are essential tools for evaluating knee instability. However, these maneuvers are inherently subjective and limited in their ability to provide quantitative data. Instrumented physical examination using an arthrometer, although capable of quantifying anterior tibial translation, does not necessarily reproduce the functional instability experienced by patients during daily activities, as it does not account for variables that significantly influence knee loading under orthostatic stress and cannot be replicated during a standard physical examination—such as individual muscle strength, body weight, and bony morphology, particularly the tibial slope, which affects articular loading under weight-bearing conditions. Thus, in the context of ACL instability, imaging the knee under weight-bearing conditions allows clinicians to observe anterior tibial translation and femorotibial rotation under physiologic standing stress, more accurately reproducing the instability experienced by patients and capturing biomechanical factors that cannot be assessed through physical examination alone. In this context, the use of WBCT in the evaluation of ACL-injured patients has been increasingly investigated in recent years (Figure 6).

A preliminary study investigated the feasibility of using WBCT to objectively quantify anterior tibial translation (ATT) and femorotibial rotation (FTR) in patients with chronic ACL tears (36). The acquisition protocol involved imaging both knees in four standardized positions under full weight bearing: bilateral knee extension, bilateral knee flexion at 30°, single-leg stance with 30° of knee flexion and internal rotation, and single-leg stance with 30° of knee flexion and external rotation. A standardized 30° knee flexion was confirmed with a goniometer, and 45° of trunk rotation to each side was applied during single-leg stance with the foot fixed, inducing internal and external rotation of the knee. Six total acquisitions per patient were performed, including single-leg stance images on both sides, with automated reconstruction occurring simultaneously with acquisition, resulting in a total procedure time of approximately 15 min. Across all positions, ACL-deficient knees demonstrated consistently higher ATT and FTR values compared with contralateral healthy knees, confirming the sensitivity of WBCT for detecting both anteroposterior and rotational instability under physiologic load (36).

**Figure 7:**
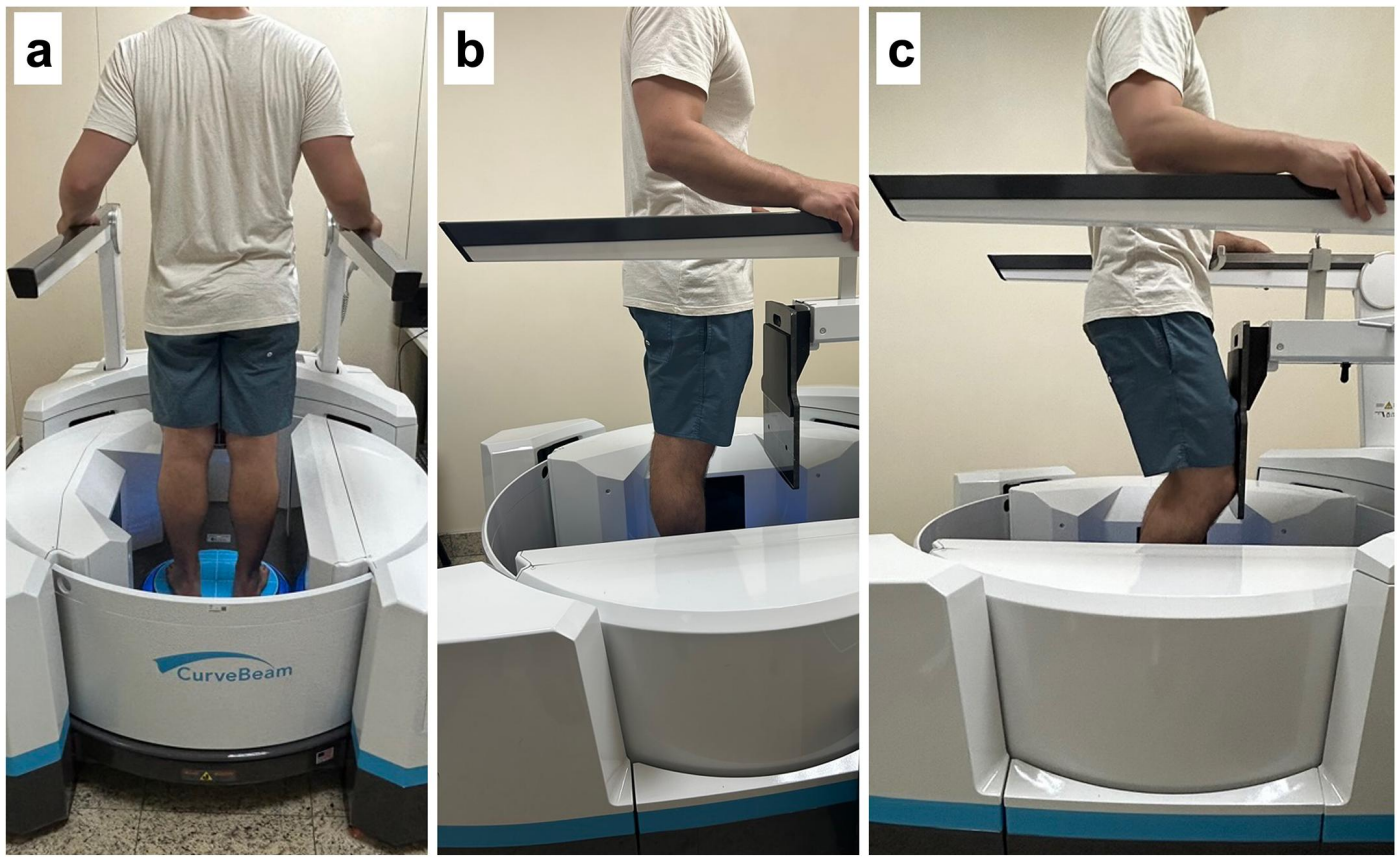
WBCT acquisition protocol for patients with anterior cruciate ligament injury (curveBeam LineUP scanner). **(a)** Posterior view of the patient standing with the knee in full extension in the cone-beam CT scanner. **(b)** Lateral view of the patient standing with the knee in full extension in the cone-beam CT scanner. **(c)** Lateral view of the patient standing with the knee flexed to 30° in the cone-beam CT scanner. Informed consent for publication of the image was obtained from the patient.

In a subsequent study, Zelada et al. (37) evaluated 20 patients with chronic unilateral ACL tears, comparing anterior tibial translation (ATT) and tibiofemoral rotation (TFR) between the injured and contralateral uninjured knees to assess the effectiveness of WBCT in detecting ACL-related instability. WBCT scans were obtained using the previously standardized bipodal positions (full extension and 30° flexion) and unipodal positions with 30° flexion and internal/external rotation. In bipodal WBCT at 30° flexion, injured knees demonstrated significantly greater lateral ATT (7.9 ± 3.8 mm vs. 4.7 ± 2.0 mm, *p* = 0.001) and medial ATT (2.9 ± 2.9 mm vs. 0.8 ± 1.4 mm, *p* = 0.007), along with increased internal TFR (10.9° ± 6.3° vs. 7.6° ± 4.6°, *p* < 0.001) compared with uninjured knees. In full extension, similar significant differences were observed, with injured knees exhibiting greater lateral ATT (2.9 ± 3.5 mm vs. 0.3 ± 0.9 mm, *p* = 0.002), medial ATT (4.6 ± 2.6 mm vs. 2.3 ± 2.5 mm, *p* < 0.001), and TFR (5.1° ± 6.3° vs. 2.1° ± 5.5°, *p* = 0.011). Under unipodal loading with flexion and either internal or external rotation, only lateral ATT remained significantly elevated in injured knees (*p* = 0.030 and *p* = 0.004, respectively), while all other parameters failed to reach statistical significance. These findings suggest that, for future studies, the protocol may be reduced to bipodal extension and 30° flexion without loss of diagnostic value (Figure 7). All measurements demonstrated good to excellent intra- and inter-observer reliability (ICC > 0.7) (37).

**Figure 8:**
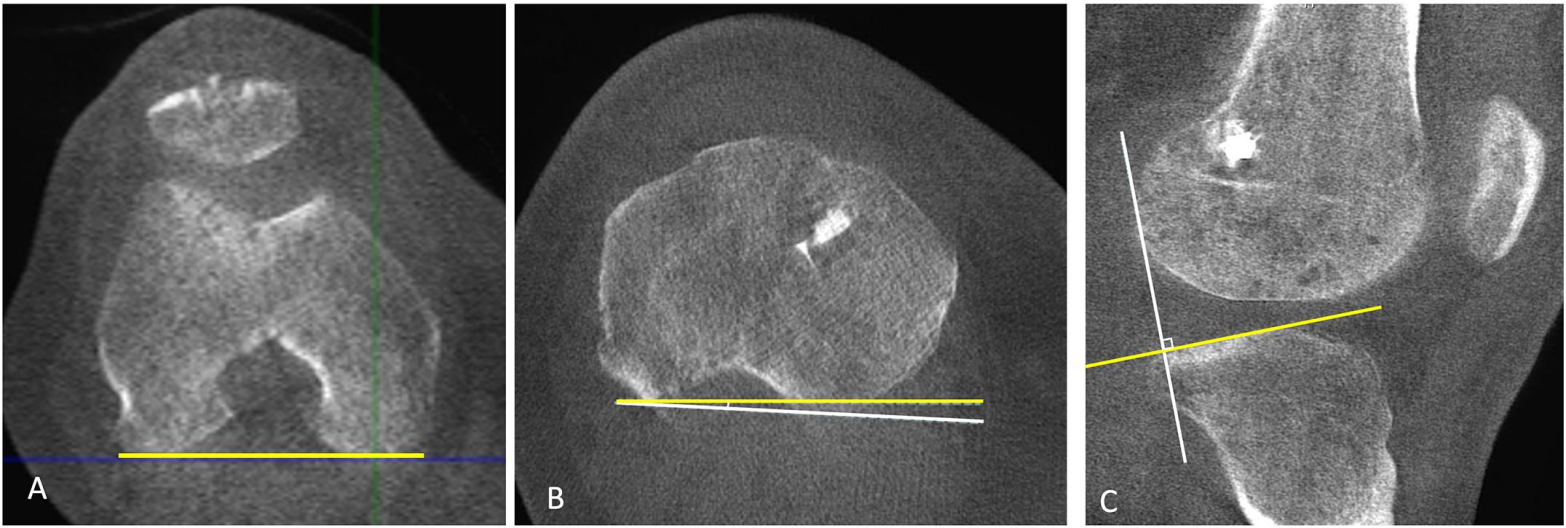
Weight-bearing CT 12 months after anatomic ACL reconstruction using interference screws for femoral and tibial fixation. Axial WBCT images **(A,B)** demonstrate external tibial rotation relative to the femur, consistent with restored rotational stability, and the sagittal WBCT image **(C)** demonstrates a negative anterior tibial translation, indicating restoration of anterior–posterior stability. The position and diameter of the tibial tunnel can be assessed on the axial image **(B)**, while the femoral tunnel can be evaluated on the sagittal image **(C)**.

Overall, the evidence to date indicates that WBCT provides a reliable and objective method for detecting both translational and rotational instability in ACL-deficient knees under physiological loading, supporting its potential role in the quantitative assessment of ACL insufficiency in clinical practice. These findings may also extend to postoperative evaluation, where WBCT could be used not only to assess correction of anterior–posterior and rotational instability, but also to evaluate tunnel positioning and overall alignment (Figure 8). Future studies should evaluate the correlation between instability measured on WBCT and findings from the physical examination, compare WBCT-derived measurements with those obtained from conventional non–weight-bearing CT or MRI, and investigate how factors such as tibial slope and body weight may influence standing stress during WBCT acquisition.

### Assessment of knee alignment and deformities

In routine clinical practice, preoperative planning for total knee arthroplasty and realignment osteotomies has long relied on two-dimensional (2D) parameters obtained from full-limb radiographs, including the hip–knee–ankle angle (HKA), lateral distal femoral angle (LDFA), medial proximal tibial angle (MPTA), joint line obliquity (JLO), and joint line convergence angle (JLCA). However, the accuracy of these measurements is highly dependent on precise radiographic acquisition, which can be compromised by suboptimal limb positioning or the presence of deformities (1, 38). Even small degrees of femoral or tibial rotation or flexion during weight-bearing radiographs can distort 2D projections and alter alignment parameters, raising concerns about reliability of radiograph-based assessments (38).

**Figure 9:**
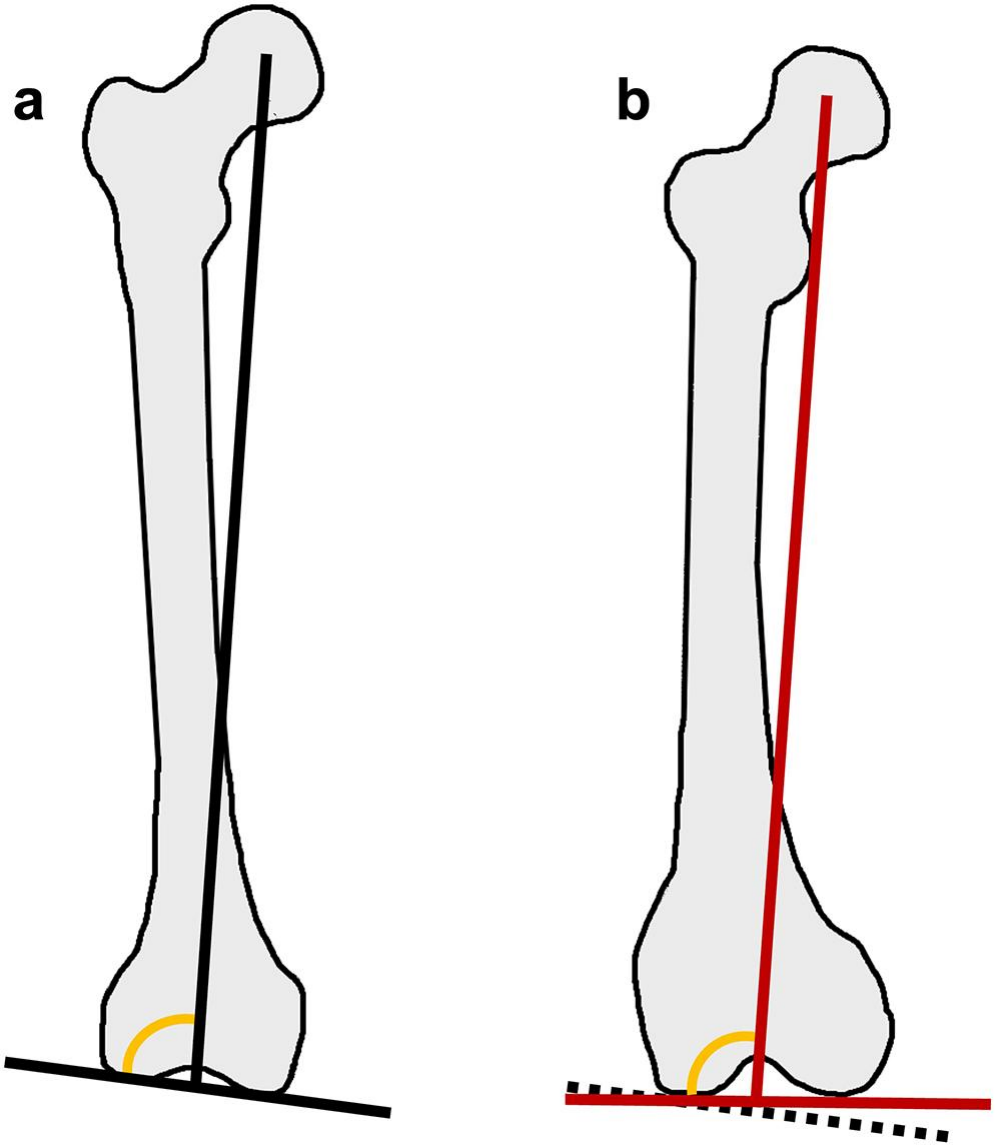
Projectional distortion related to femoral flexion and external rotation, causing an artificial reduction of the lateral distal femoral angle (LDFA). **(a)** Neutral positioning. **(b)** Femoral positioning in flexion and external rotation. Yellow: Lateral Distal Femoral Angle (LDFA).

The use of CT not only enables accurate assessment of rotational deformities in the axial plane and flexion–extension deformities in the sagittal plane, but also helps mitigate many of the projection-related errors that affect coronal plane evaluation (Figure 9). However, conventional CT performed in the supine position introduces important considerations when assessing coronal alignment. Coronal parameters such as HKA and, especially, the JLCA are load-dependent (39) and influenced by soft-tissue laxity, joint-space narrowing, and ligamentous tension that manifest only under physiological weight bearing. Therefore, NWBCT may underestimate true coronal malalignment and fail to capture the functional joint-line behavior that contributes to the overall alignment pattern (40, 41). In patients with elevated BMI and advanced osteoarthritis (KL grades 3 and 4), this discrepancy in alignment between weight-bearing and non–weight-bearing conditions may be even more pronounced (42). To overcome these limitations, intensity-based 3D/2D registration algorithms have been developed to integrate weight-bearing full-limb radiograph information with NWBCT models, allowing transformation of the CT-derived 3D anatomy into a simulated weight-bearing state and thereby enabling more standardized and reliable realignment osteotomy planning (43).

The WBCT, which provides three-dimensional imaging under physiological load and thereby overcomes many of the limitations of radiographs and conventional CT, is increasingly being investigated as a valuable tool for assessing lower-limb alignment. Comparing standing full-limb radiography and upright WBCT from patients with varus knee osteoarthritis, Sasaki et al. (44) found no correlation between the 3D joint surface orientation relative to the floor measured on WBCT and 2D coronal joint-line parameters from plain radiographs [tibial joint line angle (TJLA), MPTA, LDFA, and JLCA]. Although traditional 2D parameters were strongly correlated with one another, the absence of any relationship with the 3D measure indicates that 2D radiographs, while internally consistent, do not capture the true spatial orientation of the joint surfaces under physiological load as revealed by WBCT. In a subsequent study, Sasaki et al. (45) evaluated 66 varus knees and reported that full-limb radiographs systematically overestimated the LDFA compared with upright WBCT (2D-LDFA: 87.9° ± 2.5° vs. 3D-LDFA: 86.7° ± 3.3°; *p* < 0.05). Flexion deformity may induce relative external rotation of the femur with respect to the tibia (via the screw-home mechanism), and when the femur flexes and externally rotates, a projectional distortion occurs on radiographs: the femoral head shifts medially relative to the knee center, leading to an artificial increase in the measured LDFA. Although the MPTA showed no significant difference between 2D and 3D measurements, discrepancies in LDFA alone were sufficient to cause substantial variation in CPAK (Coronal Plane Alignment of the Knee) alignment classification (46), which describes the distribution of constitutional coronal alignment based on the arithmetic HKA and joint-line obliquity and is increasingly used to guide personalized treatment strategies. The overall agreement between 2D and 3D CPAK classifications in their study was only 48.5%, raising concerns about the reliability of CPAK classification when based solely on conventional full-limb imaging (45).

### Postoperative assessment of total knee arthroplasty

**Figure 10:**
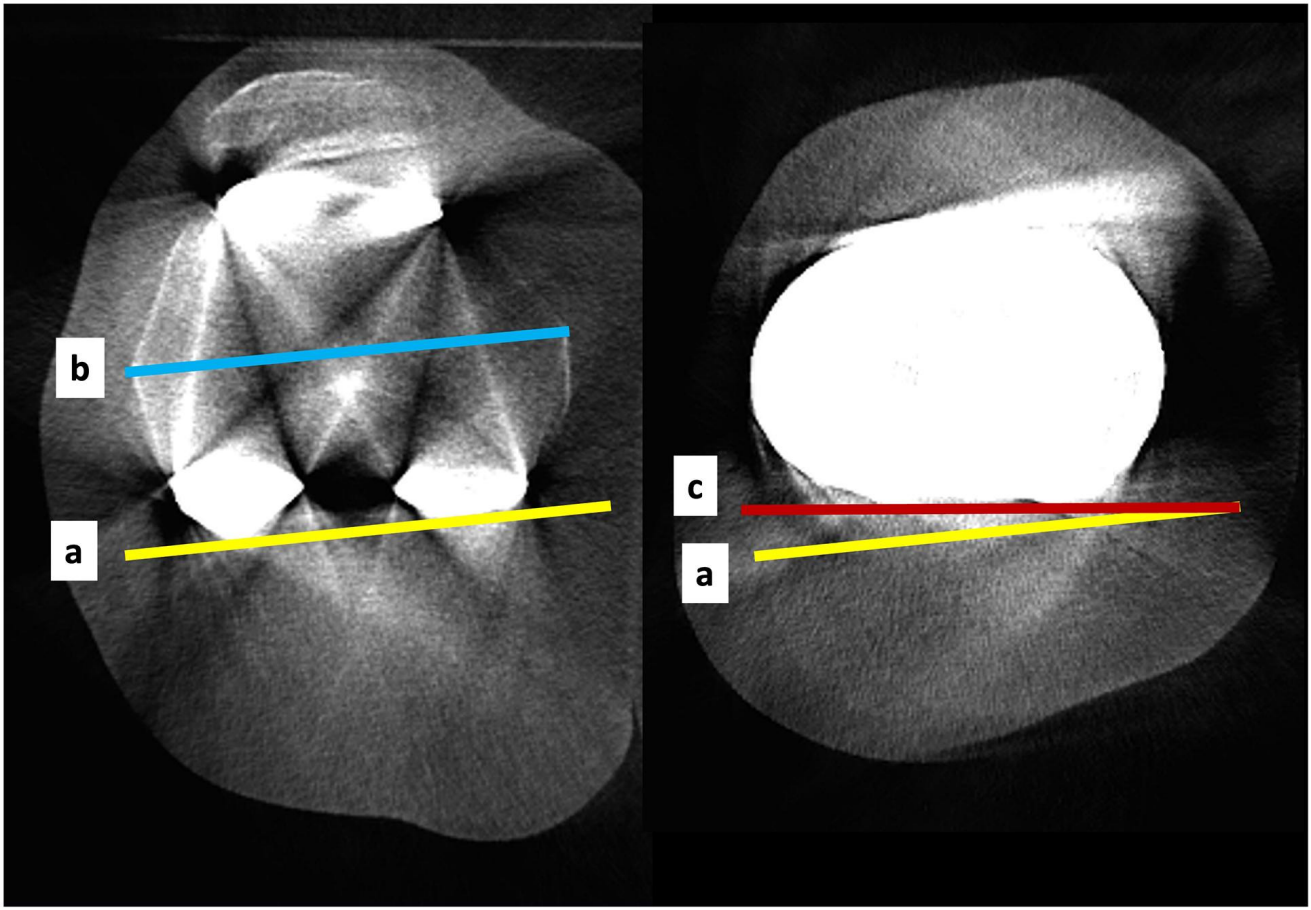
WBCT images with the knee in full extension. a: posterior femoral condylar axis; b: transepicondylar axis; c: posterior tibial condylar axis; a × c: tibiofemoral rotation angle under weight-bearing.

**Figure 11:**
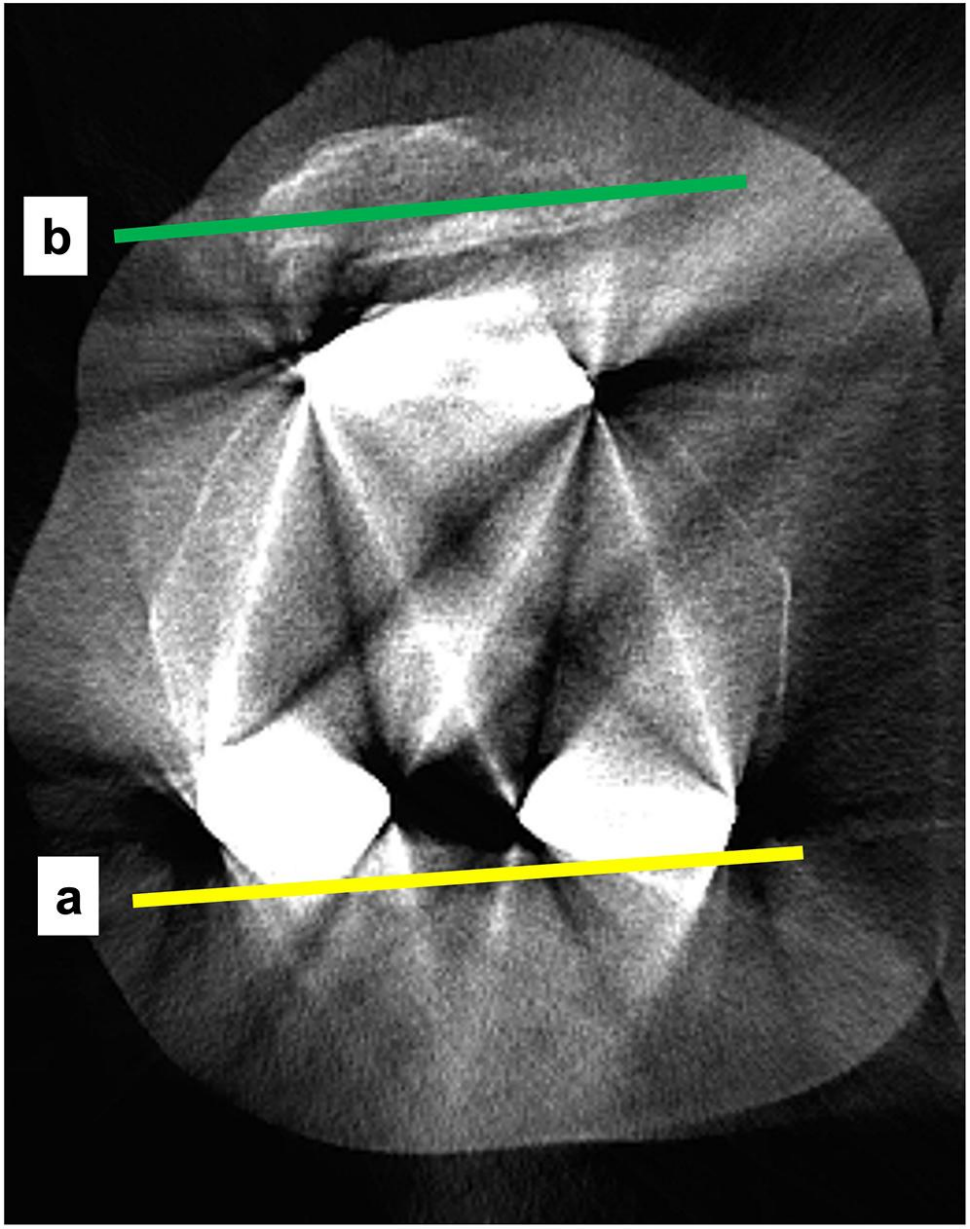
WBCT image with the knee in 30 degrees of flexion. a: posterior femoral condylar axis; b: line through the greatest width of the patella; a × b: patellar tilt angle under weight-bearing.

Another potential application of WBCT that remains underexplored in the literature is its use in the assessment of total knee arthroplasty (TKA), providing three-dimensional evaluation of the limb and the implant under physiological loading conditions (Figures 10, 11).

In relation to the usefulness of WBCT for the assessment of implant positioning, Lin et al. (47) evaluated 42 cementless TKAs approximately three years postoperatively to measure alignment, component rotation, and peri-implant bone density. WBCT showed moderate correlation with radiographs for MPTA (r = 0.555, *p* = 0.001), HKAA (r = 0.328, *p* = 0.034), and JLO (r = 0.405, *p* = 0.008), and demonstrated good to excellent repeatability for key parameters (ICC 0.84–0.97). WBCT also detected region-specific variations in bone density, such as significantly lower femoral anterior density (*p* < 0.0001) and reduced tibial anterolateral and keel density compared with posterior regions (*p* < 0.05–0.001). Overall, WBCT provided reliable, physiologically relevant postoperative assessment of component alignment and bone quality, supporting its potential role in monitoring cementless TKA fixation and early signs of loosening.

Regarding tibial implant positioning, Sasaki et al. (48) evaluated forty-three healthy knees using upright NWBCT and upright WBCT scans to determine how physiological loading affects the rotational position of the tibial anteroposterior (AP) axis—an important landmark for tibial component alignment in TKA. The AP axes defined in each condition were compared with the traditional supine-based AP axis (Akagi's line) (49), which passes through the center of the posterior cruciate ligament and the medial border of the patellar tendon attachment and was originally established as a reference line perpendicular to the femoral transepicondylar axis based on supine, non–weight-bearing CT imaging. Under upright weight-bearing, the tibial AP axis showed a mean internal rotation of 7.4° ± 4.3° relative to the traditional axis (*p* < 0.001), corresponding to a 2.9 ± 1.6 mm medial shift at the tibial plateau edge. Upright non–weight-bearing AP axis also demonstrated internal rotation relative to the traditional axis, but to a lesser degree (3.5° ± 4.1°, *p* < 0.001), with weight-bearing adding an additional 3.9° ± 4.1° of internal rotation (*p* < 0.001) (41). These findings indicate that the tibial AP axis shifts substantially under physiological loading, suggesting that the traditional supine-based Akagi's line may not fully represent functional tibial orientation during standing or gait. The identification of a consistent, weight-bearing AP axis—approximately 7.4° internally rotated, or about one-seventh of the tibial tuberosity width from the medial border—offers a practical landmark for tibial component positioning in TKA (48). Given the association between tibial malrotation and patellofemoral maltracking, altered kinematics, and implant failure, integrating weight-bearing rotational anatomy may improve component alignment, implant longevity and long-term outcomes.

In the context of evaluating TKA implant stability and aseptic loosening, Hext et al. (50) assessed the use of weight-bearing CT–based radiostereometric analysis (WBCT-RSA) compared with conventional model-based RSA (MBRSA), which depends on the intraoperative placement of invasive tantalum markers, to measure inducible displacement—the micromotion of implants between unloaded and loaded positions—as an indicator of component fixation. The study included 17 patients from a prior RSA cohort, all 5 years post-TKA, who underwent both weight-bearing and non–weight-bearing examinations with the two methods. WBCT-RSA demonstrated comparable or superior precision to MBRSA across nearly all axes of translation and rotation, and the inducible displacement values obtained with WBCT-RSA were significantly lower and remained well below established thresholds associated with implant loosening. These findings indicate that WBCT-RSA can reliably detect small, physiologically relevant micromotions without the need for invasive markers, supporting its potential as a practical, non-invasive alternative for postoperative assessment of TKA implant stability.

Although the literature on the use of WBCT for TKA assessment remains limited, the evidence to date demonstrates its ability to provide accurate and repeatable evaluations of implant alignment and rotation, bone quality, and implant stability under physiological loading, offering clinically relevant information that may enhance surgical planning, improve the detection of malposition or early loosening, and support more individualized follow-up strategies.

## Future perspectives

Weight-bearing CT represents an emerging frontier in the imaging assessment of knee pathologies. Although still a relatively recent technology, the number of studies investigating its clinical applications has grown steadily over the past decade—initially focused on the foot and ankle and, more recently, extending to the knee. Current evidence supports its utility in the evaluation of knee osteoarthritis, patellofemoral disorders, malalignment syndromes, ACL instability, and postoperative TKA assessment (Table 1), while many potential applications remain largely unexplored. In parallel with the growing emphasis on personalized treatment strategies in orthopedics, advances in biomechanical assessment, an improved understanding of constitutional alignment phenotypes and joint line obliquity, and the increasing adoption of individualized alignment philosophies, the ability to evaluate the joint with three-dimensional imaging under true physiological loading offers a wide range of new opportunities.

**Table 1:** Summary of weight-bearing CT (WBCT) evidence for the assessment of knee pathologies and their clinical significance.

Knee condition	WBCT parameters	WBCT findings	Key references
Patellofemoral instability	TT–TG distance, patellar tilt angle, congruence angle, bisect offset index, patellar height indices (Insall–Salvati, Caton–Deschamps), femorotibial rotation	Under weight bearing, TT–TG distance, patellar tilt, congruence angle and patellar height indices generally decrease compared with supine CT; bisect offset index increases; Internal tibial rotation increases and TT-TG distance decreases with flexion under weight-bearing	(13–16, 18–21)
Knee osteoarthritis	Joint space width (JSW), 3D joint space mapping,osteophytes, subchondral cysts, Meniscal extrusion distance under load alignment under load	Narrower JSW in WBCT compared to conventional CT and standing radiographs; increased meniscal extrusion than conventional CT or MRI in OA patients; higher sensitivity and accuracy than fixed-flexion radiographs for identifying osteophytes and subchondral cysts; superior diagnostic performance versus lateral radiographs for detecting patellofemoral osteoarthritis features; increased internal tibial rotation under weight-bearing correlating with OA severity; earlier detection of bone-on-bone contact	(7, 23–27, 29–32, 34, 35)
ACL instability	Anterior tibial translation (ATT) in the lateral and medial compartments; femorotibial rotation (FTR).	ACL-deficient knees demonstrate increased medial and lateral ATT and greater internal FTR compared with contralateral intact knees during bipodal stance, both in full extension and at 30° of knee flexion.	(36, 37)
Lower-limb malalignment	3D HKA, LDFA, MPTA, JLCA, joint surface orientation relative to the floor	WBCT provides true 3D alignment under physiological load, improving deformity characterization and surgical planning for osteotomies and arthroplasty	(39–46)
Total knee arthroplasty (TKA)	Component alignment and rotation, tibial AP axis, peri-implant bone density, inducible displacement (WBCT-RSA)	Weight-bearing induces internal rotation of tibial AP axis (Akagi's line); region-specific bone density changes; detectable micromotion below loosening thresholds	(47, 48, 50)

Looking ahead, the integration of WBCT with emerging technologies is likely to further expand its clinical relevance. Artificial intelligence and machine learning algorithms may facilitate automated three-dimensional measurements, alignment analysis, and the detection of subtle pathological patterns, thereby reducing examiner variability and interpretation time. WBCT datasets also hold promise for 3D printing applications, including surgical planning models and implant templating. In addition, the integration of WBCT with computer-assisted surgical navigation and robotic systems may enable more accurate, functionally informed alignment strategies in procedures such as osteotomies and TKA, effectively bridging the gap between preoperative imaging and intraoperative execution.

From a practical standpoint, future research should also focus on clinical translation and implementation pathways. A major barrier to broader clinical adoption of WBCT is the lack of standardized acquisition and measurement protocols (4). Patient positioning is a critical determinant of measurement reliability and reproducibility, with factors such as knee flexion angle, knee rotation, adduction/abduction (i.e., distance between the feet), and single- vs. double-leg stance influencing parameters relevant to the assessment of knee disorders. The literature to date reports heterogeneous protocols, including variable patient positioning strategies, different methods for controlling or measuring knee flexion (e.g., goniometers, positioning frames such as the Synaflexer™), and non-uniform approaches to image analysis. The development of pathology-specific, consensus-driven acquisition protocols, along with standardized measurement techniques and the establishment of normative reference values under weight-bearing conditions, represents an essential next step to enable reproducibility and routine clinical use. Subsequently, barriers to adoption will also include global access to the technology, reimbursement and insurance coverage, the need for dedicated technician and clinician training, and workflow integration. As these challenges are progressively addressed, WBCT has the potential to become an important tool in the comprehensive, functional assessment of knee disorders.

## Conclusion

Weight-bearing CT is emerging as a three-dimensional imaging modality that bridges the longstanding gap between static imaging and the functional, load-dependent biomechanics of the knee, offering a more comprehensive understanding across a broad range of clinical scenarios, including patellofemoral disorders, osteoarthritis, ligamentous instability, lower-limb malalignment, and postoperative follow-up.
